# RF-Phos: A Novel General Phosphorylation Site Prediction Tool Based on Random Forest

**DOI:** 10.1155/2016/3281590

**Published:** 2016-03-15

**Authors:** Hamid D. Ismail, Ahoi Jones, Jung H. Kim, Robert H. Newman, Dukka B. KC

**Affiliations:** ^1^Department of Computational Science and Engineering, North Carolina Agricultural and Technical State University, Greensboro, NC 27411, USA; ^2^Department of Electrical and Computer Engineering, North Carolina Agricultural and Technical State University, Greensboro, NC 27411, USA; ^3^Department of Biology, North Carolina Agricultural and Technical State University, Greensboro, NC 27411, USA

## Abstract

Protein phosphorylation is one of the most widespread regulatory mechanisms in eukaryotes. Over the past decade, phosphorylation site prediction has emerged as an important problem in the field of bioinformatics. Here, we report a new method, termed Random Forest-based Phosphosite predictor 2.0 (RF-Phos 2.0), to predict phosphorylation sites given only the primary amino acid sequence of a protein as input. RF-Phos 2.0, which uses random forest with sequence and structural features, is able to identify putative sites of phosphorylation across many protein families. In side-by-side comparisons based on 10-fold cross validation and an independent dataset, RF-Phos 2.0 compares favorably to other popular mammalian phosphosite prediction methods, such as PhosphoSVM, GPS2.1, and Musite.

## 1. Introduction

Protein phosphorylation, mediated by protein kinases, is one of the most important posttranslational modifications in eukaryotes. By modulating protein function via the addition of a negatively charged phosphate group to a serine (Ser, S), threonine (Thr, T), or tyrosine (Tyr, Y) residue, phosphorylation regulates many cellular processes, including signal transduction, gene expression, cell cycle progression, cytoskeletal regulation, and apoptosis [1].

It is estimated that at least 30% of the proteins in the human proteome are regulated by phosphorylation. Traditionally, phosphorylation sites in proteins have been identified using experimental techniques, such as tandem mass spectrometry (MS/MS) [2]. For instance, in a classic study, MS/MS was used to map the phosphoproteome of nine different mouse tissues, identifying 36,000 distinct phosphorylation sites [3]. Indeed, MS/MS-based approaches have yielded a wealth of information about phosphoproteomes. Nonetheless, there are various technical challenges that make identification of phosphorylation sites using MS/MS-based approaches difficult [4]. For instance, low abundance cellular proteins and proteins that are only transiently phosphorylated are often missed using MS/MS-based techniques [2]. Moreover, MS/MS-based identification is very expensive and labor intensive and requires specialized equipment and technical knowledge. In this regard, phosphosite prediction algorithms, which predict whether a residue-of-interest is likely to be phosphorylated under cellular conditions, represent potentially valuable tools for annotating the entire phosphoproteomes of a wide variety of species.

With the advent of next generation sequencing technologies, the development of accurate phosphorylation site prediction tools has become exceedingly important. As a consequence, several computational tools for the prediction of phosphorylation sites have recently been developed [5–26]. Hjerrild and Gammeltoft [6] provide an excellent overview of both the computational and biological aspects of phosphoproteomics while two recent reviews by Trost and Kusalik [5] and Xue et al. [7] summarize phosphorylation site databases, various prediction tools, and challenges associated with computational phosphorylation site prediction.

Phosphorylation site prediction can be broadly divided into two classes: kinase-specific phosphorylation site prediction and general (i.e., non-kinase-specific) phosphorylation site prediction. While kinase-specific methods aim to predict both the site of phosphorylation and the cognate kinase mediating the phosphorylation event, general phosphosite prediction methods are designed to identify putative sites of phosphorylation irrespective of the kinase. Results of the latter are analogous to those obtained by MS/MS-based experiments. Importantly, kinase-specific methods are often restricted to predictions for a relatively small subset of kinases. This is likely due, in part, to the fact that the cognate kinase is known for less than 3% of the phosphorylation sites annotated to date, severely limiting the information needed to train the algorithms [19]. Moreover, until recently, the consensus phosphorylation sites upon which kinase-specific methods rely were not available for the majority of human kinases [19]. Therefore, general phosphorylation site prediction methods offer distinct advantages when the primary goal is to predict whether or not a given site is phosphorylated [20].

Many existing general phosphorylation methods use attributes based on protein features and biological observations. For instance, DISPHOS [27], one of the first general phosphosite prediction algorithms developed, uses both position-specific amino acid frequencies and disorder information to predict sites of phosphorylation. The notion that the degree of disorder may be an important determinant of phosphorylation was based on the observation that a high percentage of cell signaling and cancer-associated proteins are predicted to have long, disordered regions [28].

Because most protein kinases catalyze the phosphorylation of a given S/T/Y residue when the residues surrounding the phosphosite fit a specific, yet flexible, pattern [29], various machine learning tools, such as artificial neural networks (ANNs) and support vector machines (SVMs), have also been used to capture the complex and subtle patterns surrounding the phosphorylated residues for phosphorylation site prediction [8, 9, 21, 22]. For instance, the general phosphosite predictor, Musite, integrates several parameters, including local sequence similarities of known phosphorylation sites, protein disorder scorers, and amino acid frequencies, as features to train a support vector classifier [26]. Likewise, both the general phosphosite prediction methods developed by Swaminathan et al. [21] and Biswas et al. [20] combine SVMs with position-specific scoring matrices (PSSMs) to identify putative phosphosites. In the case of the Swaminathan method, the authors augment their sequence-derived PSSMs with NMR-based solvent accessibility values and secondary structure predictions. Meanwhile, the Phosphorylation PREDictor (PPRED) method developed by Biswas et al. uses PSSMs based on evolutionary conservation of Ser, Thr, and Tyr residues to inform their SVMs. Finally, PhosphoSVM, which is perhaps the most robust general phosphosite prediction tool developed to date, combines eight different sequence-level scoring functions using SVMs [22]. While these methods have shown promise as general phosphosite prediction tools, methods based on ANNs, and sometimes SVMs, are often regarded as “black boxes” because it is difficult to determine exactly how a complex neural network or hidden Markov model reaches a particular solution [30].

In contrast, random forest- (RF-) based algorithms, which have been applied to various bioinformatics problems, are able to discriminate between features and to offer insights into the relative importance of each [31]. Indeed, feature importance is built into the RF framework. For this reason, RF classifiers have recently been applied to several phosphosite prediction methods. For example, to develop a kinase-specific predictor, Fan et al. conducted a systematic and hierarchy-specific prediction of phosphorylation sites in which kinases are clustered into hierarchical structures employing random forest as a classifier [18]. Likewise, the general phosphosite predictor PHOSFER [23] employed random forests to make phosphorylation site predictions in plants.

Despite steady progress in the field, the performance of existing general phosphosite prediction algorithms is not yet satisfactory with respect to parameters such as accuracy, specificity, and/or selectivity [5, 7, 22]. Importantly, in most cases, existing algorithms sacrifice one parameter for the other(s) (e.g., high specificity may come at the cost of low sensitivity or* vice versa*). Previously, we used random forest to integrate different combinations of 8 feature vectors to predict protein phosphorylation sites given only the primary amino acid sequence as input [24, 25]. While these methods performed as well or better than existing methods, their performance was still not ideal.

Here, we improve upon our previous methods by developing a general protein phosphorylation site prediction method that uses RF to integrate 10 distinct sequence and structure-based attributes. This method, which we term Random Forest-Based Phosphosite predictor 2.0 (RF-Phos 2.0), achieved uniformly high accuracy, specificity, and sensitivity scores using both 10-fold cross validation and an independent dataset. As a consequence, RF-Phos 2.0 compares favorably to existing state-of-the-art algorithms in its ability to accurately and efficiently predict phosphorylation sites given only the primary amino acid sequence as input.

## 2. Materials and Methods

### 2.1. Benchmark Dataset

The protein sequences with known Ser, Thr, and Tyr phosphorylation sites were downloaded from the PhosphoSVM website [22]. These sequences were originally obtained from P.ELM version 9.0 [32]. All phosphorylation sites in these sequences have been experimentally identified. The redundant sequences were removed using skipredundant [33] using a 30% cutoff. Namely, any sequence with identity more than 30% was removed to improve the prediction quality.  Table 1 shows the number of benchmark sequences and the number of known phosphorylation sites.

Different sized sequence windows (e.g., 5, 7, 9, 11, 15, 19, and 21 residues in length) were prepared with a given phosphorylation site residue in the middle of the window. Both positive windows, that is, those in which a known phosphosite is in the middle of the window, and negative windows, that is, those that have a S, T, or Y in the middle of the window but for which no phosphosites have been annotated, were included. As before, to avoid redundancy, the windows with high similarity were removed from both positive and negative windows. This was achieved using skipredundant [33] with a range of acceptable threshold percentage similarity between 0 and 20% and with a 10.0 gap opening penalty and 0.5 gap extension penalty. Once the redundant windows had been removed, features were then extracted from the remaining windows.  Table 2 shows the number of windows (for windows of size 9) corresponding to positive phosphorylation sites for each residue before and after redundancy removal and for negative windows after redundancy removal.

### 2.2. Protein Sequence Features

Sequence features are obtained by the process of feature extraction, which refers to extracting numeric information from protein sequences. The features are the values that can be used to learn the underlying model. Feature extraction is often the most critical step in determining whether the method will ultimately be successful. The features from windows of protein sequences were extracted using different amino acid descriptors. Some of the chosen descriptors were proposed by previous studies for phosphorylation site prediction, as it has been found that they contribute with varying degrees of information about the phosphosite. The descriptors implemented in this study are summarized as follows.

#### 2.2.1. Shannon Entropy (Feature 1)

Shannon Entropy (*H*) is known in information theory as a measure of randomness and diversity of a set of objects distributed into a space. It was defined by Shannon as a unique function that represents the average amount of information for a set of objects according to their probabilities [34]. It has been widely used in bioinformatics to score residue conservation [35]. However, in this study, instead of using position-specific entropy, which is calculated with position-specific scoring matrix (PSSM) [36], we used window-wise entropy that is calculated with probabilities of the individual amino acids in the window to generate one numeric feature. It can be calculated as

(1) $$\begin{array}{c} H=-\overset{20}{\underset{i=1}{\sum }}p_{i}\log_{2}\left(\;p_{i}\;\right), \end{array}$$

where *p*_*i*_ is the probability of an amino acid *i* = (A, C, E, D, G, F, I, H, K, M, L, N, Q, P, S, R, T, W, V, Y) in the sequence and it is computed as the total number of amino acids *i* divided by the length of the window assuming that the probability of any amino acid that does not exist in the window is zero. Entropy ranges between zero, where only one type of residue in the entire sequence is found, and 3.17, where all types of amino acids have equal occurrence in the window.

#### 2.2.2. Relative Entropy (Feature 2)

The window-wise relative entropy (RE) of two distributions *p*_*i*_ and *p*_0_, also known as Kullback-Leibler distance, is calculated as

(2) $$\begin{array}{c} RE=\overset{20}{\underset{i=1}{\sum }}p_{i}\log_{2}\left(\;\frac{p_{i}}{p_{0}}\;\right), \end{array}$$

where *p*_0_ = 1/9 is the uniform distribution of the amino acid occurrence.

RE is always nonnegative and becomes zero if and only if *p*_*i*_ = *p*_0_. As entropy, the RE is represented by one feature for each window. We again assumed that the probability of any amino acid that does not exist in the window is zero. The RE was used in previous studies to identify the conserved position [37, 38].

#### 2.2.3. Information Gain (Feature 3)

Information gain (IG) can be computed by subtracting RE from entropy. It can measure the transformation of information from the background or random state to the state influenced by the class whether the sequence is positive or negative. IG is given by

(3) $$\begin{array}{c} IG=H-RE. \end{array}$$

#### 2.2.4. Solvent Accessible Surface (ASA) (Features 4–12)

The amino acids of a protein sequence can be either buried or exposed based on their position in the 3-dimensional structure of the protein. Usually, the buried residues do not undergo posttranslational modification because they are not expected to interact with the modifying enzymes. Therefore, phosphorylation sites in the protein are expected to be exposed amino acids. Rvp-net [39], software for prediction of ASA, was used to extract ASA features from the benchmark protein sequences. ASA features were predicted before dividing the sequences into windows.

#### 2.2.5. Overlapping Properties (Features 13–102)

Overlapping properties (OP) capture the common physicochemical properties shared by the amino acids in the protein sequence [22, 40]. The amino acids were classified based on ten physicochemical properties: polar (NQSDECTKRHYW), positive (KHR), negative (DE), charged (KHRDE), hydrophobic (AGCTIVLKHFWYM), aliphatic (IVL), aromatic (FYWH), small (PNDTCAGSV), tiny (ASGC), and proline (P). An amino acid may fall into more than one group (i.e., be overlapping). Each amino acid was encoded with 10-bit, where each bit in the 10-bit code represents a group, respectively. The position of the bit is set to 1 if the amino acid belongs to the corresponding group and 0 if it does not. For example, histidine (H) is encoded with 1101101000, which indicates that it belongs to polar, positive, charged, hydrophobic, and aromatic groups. The number of features extracted with this method is *n* × 10 where *n* is the window size [40]. For the sequence window of size 9, the number of features is 90.

#### 2.2.6. Average Cumulative Hydrophobicity (Features 103–106)

The average cumulative hydrophobicity (ACH) has been used in previous studies as a protein descriptor to predict phosphorylation sites [22, 41]. ACH quantifies the tendency of the amino acids that surround the phosphorylation sites to interact with solvents. The* Eisenberg* hydrophobicity scales [42] have been used where

 A: 0.62, C: 0.29, D: −0.90, E: −0.74, F: 1.19, G: 0.48, H: −0.40, I: 1.38, K: −1.50, L: 1.06, M: 0.64, N: −0.78, P: 0.12, Q: −0.85, R: −2.53, S: −0.18, T: −0.05, V: 1.08, W: 0.81, Y: 0.26.

The number of ACH features depends on the size of the window. For a window of size 9 the ACH is computed by averaging the cumulative hydrophobicity indices of the amino acids around the putative phosphorylation site for the subwindows of the sizes 3, 5, 7, and 9, respectively, where S/T/Y is always in the center of the window. For example, to calculate ACH for the sequence KAGVSPHED, we need first to create the subwindows AGVSPHE, GVSPH, and VSP. Then we can calculate the feature of each window as

(4) $$\begin{array}{c} f=\frac{\sum_{i=1}^{n}P_{i}}{n}, \end{array}$$

where *n* is the subwindow size and *P*_*i*_ is hydrophobicity index for the amino acid in the position *i* in the window. For this example the number of features is four.

#### 2.2.7. Sequence Features (Features 107–286)

Sequence features (SF) [22] are another form of amino acid composition and they have been used recently with other feature types to predict phosphorylation sites. SF features are extracted by encoding each amino acid with a unique 20-bit of one position as 1 and other positions as zeros (e.g., 00100000000000000000). The number of the SF features depends on the window size. For instance, for a sequence with a window size of 9, the number of features will be 9 × 20 = 180.

#### 2.2.8. Composition, Transition, and Distribution (Features 287–433)

To extract the composition, transition, and distribution (CTD) features [43, 44], first the 20 amino acids are categorized into 3 groups based on one out of seven physicochemical properties each time. The seven amino acid properties are hydrophobicity; normalized Van der Waals volume; polarity; polarizibility; charge; secondary structures; and solvent accessibility [44]. Based on each property, the amino acids are encoded as 1, 2, or 3. For example, the sequence MVKELRTA is encoded as 33113122 based on hydrophobicity.

*Composition* is defined as the global percent for each encoded group in a sequence based on the property *p*, where *p* is any of the seven properties. There are 21 composition features (3 features for each one of the seven physicochemical properties). The composition is calculated as

(5) $$\begin{array}{c} C_{r,p}=\frac{n_{r}}{n},\;r=1,2,3, \end{array}$$

where *n*_*r*_ is the number of group codes *r* in the window and *n* is the number of amino acids in the window.

*Transition* is defined as the percent frequency with which a code (*r*) is followed by another code (*s*). Since there are three possible codes, the possible transitions are (1, 2), (1, 3), and (2, 3). The number of features is 21 (3 for each one of the seven physicochemical properties). The transition can be given as follows:

(6) $$\begin{array}{c} T_{rs}=\frac{n_{rs}+n_{sr}}{N-1}, \end{array}$$

where *N* is the length of the window.

*Distribution* is defined as the distribution of each encoded group (1, 2, and 3) in the sequence for the first, 25%, 50%, 75%, and 100% distributions of a particular property. The number of feature elements for the distribution is 105 (15 for each one of the seven physicochemical properties). The residue position is calculated by

(7) $$\begin{array}{c} R=Frequency\;\;of\;\;the\;\;group\times D, \end{array}$$

where *D* is 25%, 50%, 75%, or 100%. The distribution is then calculated by dividing *R* by the length of the sequence and multiplying by 100.

#### 2.2.9. Sequence Order Coupling Numbers (Features 434–493)

Sequence order coupling features are calculated using Schneider-Wrede chemical distance matrix [45]. For a protein window of *N* amino acids, the sequence order effect [46, 47] can be approximately computed as

(8) $$\begin{array}{c} \tau_{k}=\overset{N-k}{\underset{i=1}{\sum }}{\left(\;d_{i,i+k}\;\right)}^{2},\;k=1,2,3,\ldots ,m, \end{array}$$

where *τ*_*k*_ is the *k*th rank of the sequence order coupling number (SOCN), *m* is maximum lag, and *d*_*i*,*i*+*k*_ is the chemical distance between the residue in position *i* and position *i* + *k*. SOCN has 60 feature elements.

#### 2.2.10. Quasi Sequence Order (QSO) (Features 494–593)

The first 20 features of QSO [46, 47] are the frequencies of amino acids in the window and calculated by

(9) $$\begin{array}{c} X_{i}=\frac{f_{i}}{\sum_{i=1}^{20}f_{i}+w\sum_{d=1}^{m}\tau_{d}}, \end{array}$$

where *i* = 1,2,…, 20, *f*_*i*_ is the normalized frequency of the amino acid *i*, and *w* is a weighting factor (*w* = 0.1).

The features from 21 and upward reflect the sequence order using four physicochemical properties; hydrophobicity, hydrophilicity, polarity, and side-chain volume and the Schneider-Wrede chemical distance matrix [48]. These parameters are calculated by

(10) $$\begin{array}{c} X_{i}=\frac{w\tau_{k-20}}{\sum_{i=1}^{20}f_{i}+w\sum_{k=1}^{30}\tau_{k}}, \end{array}$$

where *k* = 21,22,…, 30, *w* is the weight = 0.1, and *τ*_*k*_ is the *k*th rank of the sequence order coupling as shown above. QSO has 100 feature elements. After extracting the features, the feature vector for each window can be represented as

(11) $$\begin{array}{c} f_{H_{1}},f_{{RE}_{2}},f_{{IG}_{3}},f_{{ASA}_{4}},\ldots ,f_{{ASA}_{12}},f_{{OP}_{13}},\ldots ,f_{{OP}_{102}},\\ f_{{ACH}_{103}},\ldots ,f_{{ACH}_{106}},\\ f_{{SF}_{107}},\ldots ,f_{{SF}_{286}},f_{{CTD}_{287}},\ldots ,f_{{CTD}_{433}},\\ f_{{SOCN}_{434}},\ldots ,f_{{SOCN}_{493}},f_{{QSO}_{494}},\ldots ,f_{{QSO}_{593}}, \end{array}$$

where the subscript numbers are the position indices of the feature (*f*) of the corresponding descriptor. The total number of features, based on 9-amino-acid window size, is 593.

### 2.3. Random Forest

Random forest (RF) [31] is a popular tree-based ensemble machine learning technique that is a highly adaptive method for high dimensional datasets. RF has been applied in many structural bioinformatics contexts, such as fold recognition [49], protein-protein interaction prediction [50, 51], and protein-RNA binding site prediction [52]. Essentially, the RF is a combination of a number of decision trees. Each tree is constructed with a bootstrap sample from the training dataset. It is composed of a root node, internal nodes, and terminal nodes (or leaves). Each node represents a feature that is selected based on a particular criterion. A node may have two branches. Each branch corresponds to a range of values for that selected feature. The leaves have no branches since they represent a terminal class. The node branching of the decision tree is performed by computing the Gini index for each feature. Then only the best feature that splits the training data into positive and negative sequences is selected to represent a node. Finally, the ranges of values that split the sequences will be chosen to form the decision rules.

Sequence windows are classified whether they are positive phosphorylation sites or negative sites by traversing the tree starting from the root node down to a leaf where the path is determined according to the outcome of the splitting condition at each node. We then determine to which outgoing branch the observed value of the given feature corresponds. The next node in the path is the one at the end of the chosen branch. We repeat the same operations for this node and traverse the tree until we reach a leaf. The classification is based on the general agreement of most decision trees rather than only one.

The Gini impurity index (GII) measures how often randomly chosen windows from the dataset would be incorrectly classified if they were randomly classified according to the distribution of the class in the subset of the training dataset based on the feature. The feature with the minimum impurity index will be selected for splitting.

#### 2.3.1. Feature Importance and Feature Selection

Since the Gini impurity for each feature is considered for splitting, then the feature importance can be estimated as the sum of the GII reduction over all nodes in which the specific feature is used to split the dataset. The overall importance of a feature is the average of its importance value among all trees in the forest [31, 53]. Only the most important features that split the data with less impurity are selected as predictors.

As the feature selection is integrated in the RF algorithm and is based on the feature importance, we used such scores to select the 100 most important features and we then used them to train our model to see whether the use of only the top 100 features introduces any improvement to the performance.

#### 2.3.2. RF Parameters

For better results, RF requires the number of trees in the forest to be optimized. To choose the best value for the number of trees, different values were evaluated and the performance was recorded each time. Then the values that contribute to the best performance were selected.

#### 2.3.3. Phosphosite Prediction

The RF is a robust learner and less prone to generalization error and overfitting. The prediction of the phosphorylation site depends on probabilistic averaging of the decision trees rather than voting for a single class. A vector of probabilities corresponding to the class will be given at each prediction process. A sequence will be assigned the most probable class, either positive or negative.

### 2.4. Model Evaluation

The goal of the model evaluation is to assess the models thoroughly for prediction performance. In this study, both 10-fold cross validation and independent test sample were used and the evaluation metrics were calculated accordingly. 

*(i) 10-Fold Cross Validation*. The 10-fold cross validation was conducted to construct and test the classification model. The windows were split randomly into ten equal partitions, from which nine partitions were used to construct the model and one was used to test the model each time repeatedly. 

*(ii) Independent Test Set*. An independent test dataset was also used to evaluate RF-Phos 2.0 and other phosphosite prediction methods. The sequences for this test dataset were also downloaded from the P.ELM database. To avoid overfitting, this dataset does not contain any sequences that are in the Benchmark Dataset. The features were extracted from the test sequences in the same way as described above. Features corresponding to a window size of 9, with positive S/T/Y in the middle of the window, were prepared as a positive dataset. A negative dataset for each residue was prepared by using features of windows with S/T/Y in the middle that are not annotated as phosphosites. Window size of 9 was chosen for subsequent analysis based on our performance calculation for various window sizes, namely, 7, 9, 11, 13, 15, 17, 19, and 21 (see Supplementary Materials for the results, available online at http://dx.doi.org/10.1155/2016/3281590). To use balanced positive and negative test datasets, a number of negative windows equal to the number of corresponding positive windows were selected randomly.  Table 3 shows the numbers of the positive and negative windows in the independent test set (86 sequences total).

#### 2.4.1. Description of Existing Phosphosite Prediction Tools

Several popular general phosphosite prediction tools designed to predict mammalian phosphorylation sites were evaluated. These methods, which are based on various learning methods, are described briefly below.* NetPhos* is a general phosphosite predictor that uses structural information as features to train an ANN [8].* Musite* integrates local sequence similarities of known phosphorylation sites, protein disorder scorers, and amino acid frequencies as features to train the support vector classifier [26]. The method developed by Swaminathan et al. uses SVMs to integrate experimentally derived solvent accessibility values and secondary structure prediction methods.* PPRED* uses SVM and PSSMs based on evolutionary conservation of S, T, and Y phosphosites to predict putative sites of phosphorylation within a protein sequence. Finally,* PhosphoSVM* is a general prediction tool that uses support vector machine (SVM) to make classification decisions that distinguish between phosphorylation and nonphosphorylation sites [22]. It combines eight amino acid properties as features to make decisions about phosphosites.

In addition to the general phosphosite prediction tools described above, we also included two popular kinase-specific tools in the comparison. It is important to note that, for the purposes of this study, we were only interested in assessing the ability of these kinase-specific tools to predict sites of phosphorylation (therefore, we were not interested in whether they correctly predicted the cognate kinase). The kinase-specific methods are described below.* NetPhosK* is a kinase-specific prediction tool that uses an artificial neural network (ANN) predictor to identify putative sites of phosphorylation based on consensus phosphorylation motifs [54].* GPS 2.1* is kinase-specific phosphorylation site prediction tool that uses motif length selection (MLS) [7] and uses an amino acid substitution matrix BLOSUM62 and then applies clustering to identify potential phosphosites.

#### 2.4.2. Evaluation Metrics

In the case of both 10-fold cross validation and the independent test set, the phosphorylation site in a test window is predicted each time and annotated as either a positive or negative site. This gives rise to four frequencies: true positive (TP), false positive (FP), true negative (TN), and false negative (FN). Those four frequencies were used to calculate the evaluation metrics for each type of evaluation. The metrics included accuracy, precision, sensitivity, specificity, *F*1 score, Matthew's correlation coefficient (MCC), and the area under the ROC curve (AUC):

(12) Accuracy=TP+TNTP+TN+FP+FN×100,Precision=TPTP+FP×100,Sensitivity=TPTP+FN×100,Specificity=TNTN+FP×100,F1  score=2×Precision×SensitivityPrecision+Sensitivity,MCC=TPTN−FPFN√TP+FPTP+FNTN+FPTN+FN.

## 3. Results and Discussion

### 3.1. RF Parameters

#### 3.1.1. The Number of Trees in the Forest

**Figure 1:**
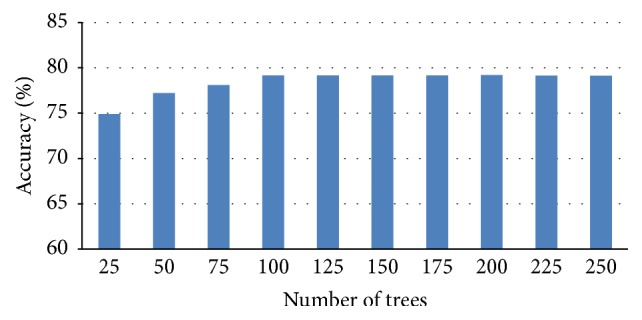
Accuracy versus number of tree for serine.

The number of trees in the random forest is an important parameter that needs to be optimized in order to obtain the best results. In order to find an optimal number of trees, we plotted the accuracy versus the number of trees for the three different types of phosphosites (Figure 1). The number of trees that achieved the greatest accuracy is 100. Importantly, the accuracy does not increase even if the number of trees is further increased beyond this number. The minimal number of trees that was found to achieve the greatest accuracy is 100.

### 3.2. Feature Importance and Feature Selection

**Figure 2:**
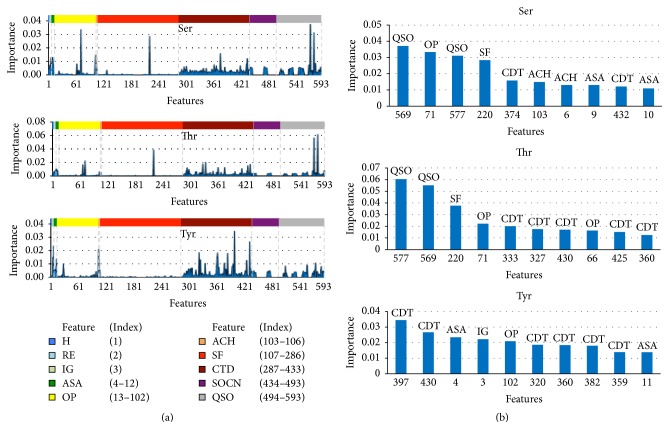
Feature distribution. (a) Distribution of the feature importance of all 593 features for Ser (top), Thr (middle), and Tyr (bottom). Features and corresponding indices are noted. Dashed lines represent boundaries between feature indices. (b) Top ten important features Ser (top), Thr (middle), and Tyr (bottom). The bar labels indicate the feature type to which the important features belong. CTD: composition, transition, and distribution; ASA: accessible surface area; SF: sequence features; ACH: average cumulative hydrophobicity; OP: overlapping properties.

In RF, Gini feature importance is implemented to estimate the feature importance. Each feature will have a weight that indicates the level of importance. Thus, the features were first indexed from 1 to 593 and the distributions of feature importance for Ser, Thr, and Tyr were determined (Figure 2(a)). While parts of several features, including Shannon entropy (*H*), relative entropy (RE), information gain (IG), quasi sequence order (QSO), and composition, transition, and distribution (CTD), appear to be important for all three residues, overlapping properties (OP) and sequence features (SF) exhibit a high degree of importance for Ser and Thr but not Tyr. Interestingly, the feature importance profiles for Ser and Thr appear to mirror one another, while that of Tyr is more divergent. This is consistent with the notion that Ser and Thr are biochemically more similar to one another than to Tyr.

To gain further insights into the molecular determinants governing phosphosite selection, next we examined the top ten features for each residue (Figure 2(b)). While only the top ten features are shown, it is important to note that other important features, not included within the top ten, might also be selected for internal node splitting in the training process. Nonetheless, this approach allowed us to observe general trends about the importance of the various features. Consistent with the overall feature distribution observed in Figure 2(a), four of the top five most important features are shared between Ser and Thr (specifically, QSO_569_, QSO_577_, SF_220_, and OP_71_). The fact that these features are all related to patterns in the sequence order and/or physicochemical properties of the amino acids is consistent with the observation that Ser/Thr kinases tend to recognize well-defined consensus phosphorylation motifs present in their substrates while Tyr kinases are generally more promiscuous.

Interestingly, the top ten feature distributions for both Tyr and Thr are dominated by CTD (6 of the top 10 for Tyr; 5 of 10 for Thr) (Figure 2(b), Table 4). This prevalence of CTD is particularly evident within the “second tier” of Thr features (features 6–10), where 4 of the 5 features correspond to CTD. Though this feature domain is less prominent among the top ten features for Ser (only 2 of 10), it is apparent from Figure 2(b) that CTD still plays an important role in phosphosite prediction. Together, these data suggest that CTD is a determining factor in improving the phosphorylation site prediction. Likewise, the high profile of QSO for Ser and Thr suggests that it is likely a determining factor in improving phosphorylation site prediction for these two residues. Interestingly, the IG, which is calculated using both Shannon entropy and relative entropy, was the fourth most important feature for Tyr.

Once the relative feature importance was determined, we next asked if the performance of our algorithm would benefit by training it with only the top 100 features instead of the full complement of 593 features. We hypothesized that, by limiting the number of features to those that are most important, we may be able to reduce the noise, leading to better predictions. Therefore, we trained the model twice. The first time, we used the entire set of 593 features, allowing the integrated feature selection to select the features that best split the data into their corresponding classes, as guided by the algorithm. The second time, we trained the model with only the top 100 features based on feature weights. A comparison of the evaluation metrics obtained during 10-fold cross validation of each model revealed that, overall, the model trained using the entire set of features performed slightly better than that trained using only the top 100 features (Table 5). For instance, in the case of Ser, the model trained using the full complement of features scored 3–6% higher in all areas than the model trained with only the top 100 features. Similar results were observed for Thr, with some notable exceptions. For instance, there was a 15.5% decrease in Thr sensitivity when the number of features used for training was reduced to 100. However, there was also a slight (4.6%) increase in specificity and a corresponding increase in the *F*1-score (2.1% increase) when the top 100 features were used. Finally, in the case of Tyr, there did not appear to be a major impact on performance since no metric varied by more than 3% in either direction. Therefore, since the overall performance of the model trained with the full complement of features appeared to be slightly better than that trained with only the top 100 features, we used the entire set of features to train RF-Phos 2.0.

### 3.3. RF Prediction Results

**Figure 3:**
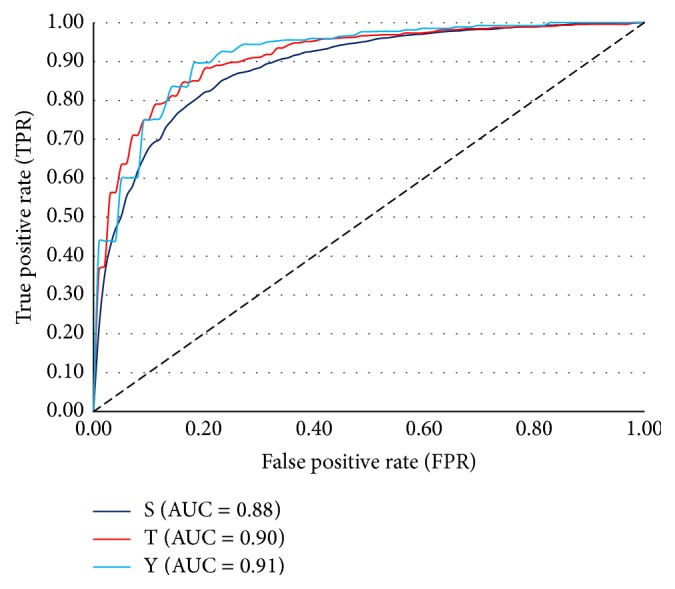
The receiver operating characteristic (ROC) curve of RF-Phos 2.0 using 10-fold cross validation.

As can be seen from the 10-fold cross validation results shown in Figure 3 and Table 5, RF-Phos 2.0 accurately predicts phosphorylation sites for the residues Ser, Thr, and Tyr, exhibiting rates of 80%, 84%, and 84% respectively. This indicates that our model is able to predict, with reasonably high confidence, whether a given site is positive or negative. Likewise, RF-Phos 2.0 achieved precision scores ranging from 81% to 85%, suggesting that it is able to identify true positives while minimizing false positives. Finally, RF-Phos 2.0 also performed well with respect to both sensitivity, which measures the percentage of positive sites that are predicted correctly out of all known positive sites (Ser: 84%; Thr: 83%; Tyr: 83%), and specificity, which measures the model's ability to correctly identify negative sites (Ser: 85%; Thr: 94%; Tyr: 88%).

Given its uniformly high scores in the above areas, it is not surprising that RF-Phos 2.0 also performed well based on composite scoring methods, such as the *F*1-score, which combines both precision and sensitivity into one score as an unbiased measure for dichotomous datasets. Indeed, during the 10-fold cross validation, RF-Phos 2.0 achieved *F*1-scores of 82%, 80%, and 82% for Ser, Thr, and Tyr, respectively.

Likewise, RF-Phos 2.0 exhibited Matthew's correlation coefficients (MCC) of 0.59, 0.67, and 0.69 for Ser, Thr, and Tyr, respectively. As a correlation index, the MCC reflects the agreement between the observation and the prediction, where 1.0 indicates perfect agreement, −1.0 means complete disagreement, and 0 is the score that can be achieved with random prediction. Therefore, the MCC scores achieved by RF-Phos 2.0 imply fairly good agreement between the observed phosphosites and those predicted by RF-Phos 2.0.

### 3.4. Comparison with Existing Methods

Next, we asked how well RF-Phos 2.0 performed relative to several existing general phosphosite prediction methods, such as NetPhos [8], Musite [26], and PhosphoSVM [22], as well as to the popular kinase-specific methods, NetPhosK [54] and GPS2.1 [7]. To this end, we compared RF-Phos 2.0 to the other methods using both 10-fold cross validation and an independent dataset generated using MS-MS data curated from P.ELM. In both cases, RF-Phos 2.0 performed very well compared to the existing methods (Tables 6 and 7). For instance, in the 10-fold cross validation, RF-Phos 2.0 exhibited the highest AUC and MCC scores among all of the methods evaluated.

It should be noted that the AUC of many of the existing methods is close to 0.5, which would be expected from random prediction alone. Likewise, the MCC of the existing methods are close to zero. In contrast, RF-Phos 2.0 exhibited AUCs ranging from 0.88 to 0.91 and MCCs between 0.65 and 0.70 (Figure 3; Table 6). This represents an approximately 25–50% improvement over existing methods with respect to AUC and an approximately 3- to 8-fold improvement over existing methods with respect to MCC.

Similar results were obtained when an independent dataset was used to compare the methods (Table 7). Importantly, RF-Phos 2.0 also exhibited sensitivity and specificity scores that were comparable to those of the highest performing methods in each category. In other words, RF-Phos 2.0 does not sacrifice sensitivity for specificity and* vice versa*. As a consequence, RF-Phos 2.0 achieved the highest MCC scores among all of the methods. This metric, which integrates information about TP, TN, FP, and FN rates, serves as a comprehensive indicator of performance. Together, these data suggest that RF-Phos 2.0 is a potentially powerful new tool for general phosphorylation site prediction.

## 4. Conclusion

We have developed a general phosphorylation site prediction method, termed RF-Phos 2.0, which uses RF to integrate various sequence and structure-based attributes to identify phosphorylation sites in proteins given only the primary amino acid sequence as input. The use of RF allowed us to calculate the relative importance of each feature (Figure 2(a)), revealing that Shannon entropy (*H*), relative entropy (RE), quasi sequence order (QSO), sequence order coupling number (SOCN), and composition, transition, and distribution (CTD) are some of the most important features for phosphorylation site prediction using our method. Among these, *H* and RE are quite different from the features used in previous phosphorylation site prediction methods. Importantly, these features do not rely on position-specific scoring matrices (PSSMs), which would impart a heavy computational cost on the algorithm. Indeed, those two descriptors, with their simple form, had a substantial effect on the predictive power of RF-Phos 2.0 (Figure 2(a)). Likewise, in this study, QSO and SOCN are used for the prediction of phosphorylation sites for the first time. As illustrated in Figure 2, both QSO and SOCN positively impact the predictive power of our model. This is particularly evident in the case of Ser and Thr. Moreover, because RF-Phos 2.0 uses RF, which is an assembly of classifiers created from bootstrap sampling of the same dataset, the prediction is more robust and not influenced by outliers compared to other machine learning methods that depend on a uniclassifier.

To evaluate our model, both a 10-fold cross validation strategy and an independent test dataset were used to calculate a comprehensive set of evaluation metrics. Compared to several existing mammalian general phosphorylation site prediction methods (e.g., NetPhos, Musite, and PhosPhoSVM) and two popular kinase-specific methods (i.e., NetPhosK and GPS 2.1), RF-Phos 2.0 performed better in overall performance (i.e., MCC) and comparably in all other areas. In addition to the factors outlined above, this may be due to the fact that we used the largest number of nonredundant sequences for training and testing among the other studies. This was done to avoid overfitting, which widens the generalization error. Recently, similar results were observed when PHOSFER, an RF-based phosphosite prediction method trained against plant phosphoproteomes, was compared to existing* Arabidopsis* phosphosite prediction tools [23].

It should be noted that, though RF-Phos 2.0 exhibited the highest MCC among all of the methods tested, the fact that it achieved scores ranging from 0.29 to 0.50 (depending on the residue) using an independent test set suggests that there is still plenty of room for improvement. In the future, we will explore other parameters (e.g., evolutionary conservation of putative phosphorylation sites) that may further improve the predictive power of our model. Likewise, kinase information may be integrated into the model. Indeed, recent studies have shown that when information exists about the kinase(s) that phosphorylate a given target protein (irrespective of the specific site(s) of phosphorylation) or when knowledge of the species- or group-specific classification of the target is known beforehand, general phosphosite prediction methods that integrate this information perform particularly well [7].

Together, this work will help annotate and mark the most probable phosphorylation sites in a protein sequence, potentially reducing the time and cost required for positive phosphosite identification using experimental methods. To facilitate its use by the signaling community, RF-Phos 2.0 is freely available at http://bcb.ncat.edu/RF_Phos/.

## Supplementary Material

The supplementary Tables (1, 2, 3, 4, and 5) show the accuracy, precision, sensitivity, specificity, F1-score, Matthew's correlation coefficient (MCC), and the area under the curve based on 10-fold cross validation for models trained with sequences of window-sizes of 7, 11, 15, 19, and 21, respectively.

## Figures and Tables

**Table 1:** The benchmark sequences of known phosphorylation sites.

Residue	Number of sequences	Number of sites
Ser	6,635	20,964
Thr	3,227	5,685
Tyr	1,392	2,163

**Table 2:** The number of windows before and after redundancy removal for size = 9.

Residue	Positive windows		Negative
	Before	After	Used
Ser	20577	1554	1543
Thr	5596	707	453
Tyr	2124	267	226

**Table 3:** Independent test set.

Residue	Positive/negative
Ser	307/307
Thr	68/68
Tyr	51/51

**Table 4:** Feature types and their count percentage in the top-ten important features for each phosphosite.

Residues	Features
S	ASA (30%), CDT (20%), QSO (20%) ACH (10%), OP (10%), and SF (10%)
T	CTD (50%), OP (20%), QSO (20%), and SF (10%)
Y	CDT (60%), ASA (20%), IG (10%), and OP (10%)

**Table 5:** Evaluation metrics obtained from 10-fold cross validation for the model trained using either the entire set of 593 features (“all”) or the top 100 features (“100”). Results using all 593 features are shown in boldface.

Metrics	Residues							
	S			T			Y	
	All	100		All	100		All	100
Accuracy	**83.00**	80.00		**87.00**	84.00		**86.00**	85.00
Precision	**84.00**	79.00		**89.00**	87.00		**86.00**	88.00
Sensitivity	**84.00**	81.00		**83.00**	87.00		**83.00**	84.00
Specificity	**85.00**	80.00		**94.00**	79.00		**88.00**	84.00
*F*1-score	**84.00**	80.00		**85.00**	87.00		**84.00**	86.00
MCC	**0.65**	0.61		**0.70**	0.66		**0.70**	0.69
AUC	**0.88**	0.85		**0.90**	0.85		**0.91**	0.88

**Table 6:** Scoring metrics using 10-fold cross validation.

Methods	Residue = S			
	AUC	Sen (%)	Sp (%)	MCC
NetPhosK	0.63	50.9	67.8	0.08
GPS 2.1	0.74	33.1	93.3	0.20
Swaminathan	0.70	31.3	88.7	0.13
NetPhos	0.70	34.1	86.7	0.12
PPRED	0.75	32.3	91.6	0.17
Musite	0.81	41.4	93.7	0.25
PhosphoSVM	0.84	44.4	94.0	0.30
**RF-Phos**	**0.88**	**84.0**	**85.0**	**0.65**

Methods	Residue = T			
	AUC	Sen (%)	Sp (%)	MCC

NetPhosK	0.60	62.0	56.8	0.07
GPS 2.1	0.70	38.1	92.3	0.20
Swaminathan	0.72	28.0	92.5	0.14
NetPhos	0.66	34.3	83.7	0.09
PPRED	0.73	30.3	91.0	0.13
Musite	0.78	33.8	94.8	0.22
PhosphoSVM	0.82	37.3	95.0	0.25
**RF-Phos**	**0.90**	**83.0**	**94.0**	**0.70**

Methods	Residue = Y			
	AUC	Sen (%)	Sp (%)	MCC

NetPhosK	0.60	39.5	74.2	0.08
GPS 2.1	0.61	34.5	78.9	0.08
Swaminathan	0.62	60.5	57.0	0.09
NetPhos	0.65	34.7	84.5	0.13
PPRED	0.70	43.0	82.7	0.17
Musite	0.72	38.4	86.7	0.18
PhosphoSVM	0.74	41.9	87.3	0.21
**RF-Phos**	**0.91**	**83.0**	**88.0**	**0.70**

**Table 7:** Scoring metrics using an independent test dataset.

Methods	Residue = S		
	Sen (%)	Sp (%)	MCC
NetPhosK	80.13	38.79	0.10
GPS 2.1	94.79	28.62	0.14
NetPhos	76.55	54.20	0.16
PHOSFER	74.59	65.51	0.22
Musite	55.70	87.39	0.31
PhosphoSVM	63.84	81.76	0.29
**RF-Phos**	**72.00**	**70.00**	**0.41**

Methods	Residue = T		
	Sen (%)	Sp (%)	MCC

NetPhosK	69.12	50.82	0.06
GPS 2.1	95.59	20.84	0.07
NetPhos	54.41	77.43	0.12
PHOSFER	77.94	64.77	0.14
Musite	48.53	93.55	0.26
PhosphoSVM	70.59	78.16	0.19
**RF-Phos**	**79.00**	**80.00**	**0.50**

Methods	Residue = Y		
	Sen (%)	Sp (%)	MCC

NetPhosK	25.49	83.23	0.04
GPS 2.1	98.04	21.42	0.09
NetPhos	64.71	67.50	0.13
PHOSFER	62.75	59.29	0.08
Musite	47.06	88.77	0.20
PhosphoSVM	82.35	64.18	0.18
**RF-Phos**	**61.00**	**62.00**	**0.29**
