# Supplementary material for: RF-Phos: A Novel General Phosphorylation Site Prediction Tool Based on Random Forest
Source: Biomed Res Int. 2016 Mar 15;2016:3281590. doi: 10.1155/2016/3281590 (PMC4811047; doi:10.1155/2016/3281590)
Supplement: Supplementary file 1 — The supplementary Tables (1, 2, 3, 4, and 5) show the accuracy, precision, sensitivity, specificity, F1-score, Matthew's correlation coefficient (MCC), and the area under the curve based on 10-fold cross validation for models trained with sequences of window-sizes of 7, 11, 15, 19, and 21, respectively. [file 3281590.f1.docx]

*Supplementary Materials for*

*RF-Phos: A Novel General Phosphorylation Site Prediction Tool*

*Based on Random Forest*

Hamid D. Ismail^1^, Ahoi Jones2, Jung H. Kim^2^, Robert H. Newman^3^*, Dukka B. KC^1^*

Departments of ^1^Computational Science and Engineering, ^2^Electrical and Computer Engineering, and ^3^Biology, North Carolina Agricultural and Technical State University, Greensboro, NC, 27411

SUPPLEMENTARY TABLE I
 EVALUATION METRICS OBTAINED FROM 10-FOLD CROSS VALIDATION USING WINDOW SIZE 7 USING ALL THE 593 FEATURES

| **Metrics** | **Residues** | | |
| --- | --- | --- | --- |
|  | **S** | **T** | **Y** |
| Accuracy | 66 | 60 | 63 |
| Precision | 67 | 57 | 55 |
| Sensitivity | 95 | 84 | 87 |
| Specificity | 95 | 28 | 25 |
| F1-Score | 79 | 71 | 74 |
| MCC | 0.10 | 0.15 | 0.15 |
| AUC | 0.53 | 0.56 | 0.56 |

SUPPLEMENTARY TABLE II
 EVALUATION METRICS OBTAINED FROM 10-FOLD CROSS VALIDATION USING WINDOW SIZE 11 USING ALL THE 593 FEATURES

| **Metrics** | **Residues** | | |
| --- | --- | --- | --- |
|  | **S** | **T** | **Y** |
| Accuracy | 85 | 86 | 74 |
| Precision | 85 | 91 | 74 |
| Sensitivity | 85 | 96 | 79 |
| Specificity | 85 | 69 | 68 |
| F1-Score | 85 | 90 | 77 |
| MCC | 0.71 | 0.70 | 0.48 |
| AUC | 0.85 | 0.82 | 0.74 |

SUPPLEMENTARY TABLE III
 EVALUATION METRICS OBTAINED FROM 10-FOLD CROSS VALIDATION USING WINDOW SIZE 15 USING ALL THE 593 FEATURES

| **Metrics** | **Residues** | | |
| --- | --- | --- | --- |
|  | **S** | **T** | **Y** |
| Accuracy | 86 | 79 | 75 |
| Precision | 89 | 79 | 76 |
| Sensitivity | 82 | 87 | 87 |
| Specificity | 82 | 68 | 71 |
| F1-Score | 85 | 0.83 | 74 |
| MCC | 0.73 | 0.57 | 0.50 |
| AUC | 0.86 | 0.75 | 0.75 |

SUPPLEMENTARY TABLE IV
 EVALUATION METRICS OBTAINED FROM 10-FOLD CROSS VALIDATION USING WINDOW SIZE 19 USING ALL THE 593 FEATURES

| **Metrics** | **Residues** | | |
| --- | --- | --- | --- |
|  | **S** | **T** | **Y** |
| Accuracy | 78 | 84 | 82 |
| Precision | 76 | 83 | 80 |
| Sensitivity | 87 | 91 | 78 |
| Specificity | 87 | 71 | 85 |
| F1-Score | 81 | 88 | 81 |
| MCC | 0.57 | 0.64 | 0.64 |
| AUC | 0.78 | 0.81 | 0.82 |

SUPPLEMENTARY TABLE III
 EVALUATION METRICS OBTAINED FROM 10-FOLD CROSS VALIDATION USING WINDOW SIZE 21 USING ALL THE 593 FEATURES

| **Metrics** | **Residues** | | |
| --- | --- | --- | --- |
|  | **S** | **T** | **Y** |
| Accuracy | 78 | 77 | 72 |
| Precision | 77 | 77 | 74 |
| Sensitivity | 91 | 91 | 81 |
| Specificity | 53 | 53 | 62 |
| F1-Score | 84 | 84 | 76 |
| MCC | 0.49 | 0.49 | 0.44 |
| AUC | 0.72 | 0.72 | 0.72 |
